# Metallo-β-lactamase–producing Enterobacterales: cyanide-containing efflux pump inhibitors as potential dual-activity inhibitors

**DOI:** 10.1093/jac/dkag122

**Published:** 2026-03-31

**Authors:** Otávio Hallal Ferreira Raro, Csaba Szabo, Patrice Nordmann

**Affiliations:** Faculty of Science and Medicine, Medical and Molecular Microbiology Unit–Department of Oncology, Microbiology, Immunology (OMI), University of Fribourg, Fribourg CH-1700, Switzerland; Faculty of Science and Medicine, Pharmacology Unit–Department of Oncology, Microbiology, Immunology (OMI), University of Fribourg, Fribourg, Switzerland; Faculty of Science and Medicine, Medical and Molecular Microbiology Unit–Department of Oncology, Microbiology, Immunology (OMI), University of Fribourg, Fribourg CH-1700, Switzerland; Faculty of Science and Medicine, Swiss National Reference Center for Emerging Antibiotic Resistance (NARA), University of Fribourg, Fribourg, Switzerland

## Abstract

**Background and objectives:**

Metallo-β-lactamase (MBL)–producing Enterobacterales represent a major public health threat because they confer resistance to all β-lactams except monobactams. The most prevalent MBLs include VIM, IMP, and NDM types, which render all clinically approved β-lactamase inhibitors ineffective. This study evaluates whether cyanide-containing molecules such as the efflux pump inhibitors carbonyl cyanide *m*-chlorophenylhydrazone (CCCP), carbonyl cyanide 4-trifluoromethoxyphenylhydrazone (FCCP) or the cytochrome C oxidase inhibitor potassium cyanide (KCN) can restore carbapenem susceptibility in MBL–producing strains by disrupting zinc-dependent enzymatic activity.

**Methods:**

A series of isogenic *Escherichia coli* TOP10 and MG1655 strains carrying the shuttle vector pUCP24 harbouring various MBLs and clinical strains were included in this study. The strains were subjected to antimicrobial susceptibility testing by broth microdilution, checkerboard assays, determination of the 50% inhibitory concentration (IC_50_) of carbapenemase activity, kinetic assays and time-kill curve assays.

**Results:**

CCCP, FCCP and KCN restored carbapenem (imipenem) susceptibility in *E. coli* strains producing VIM-1–like enzymes. Synergy was observed in checkerboard assays, and reduction of bacterial growth was confirmed by time-kill experiments. The addition of Zn^2+^ reversed this effect, supporting a Zn^2+^-chelating or Zn^2+^-displacing mechanism. Non–VIM-1-like MBLs, including VIM-2, IMP-1 and NDM-1, were not affected by the addition of cyanide-containing compounds, indicating strong selectivity of these inhibitory effects. IC_50_ measurements confirmed the selective activity of CCCP, FCCP and KCN against VIM-1–like MBLs.

**Conclusions:**

CCCP, FCCP and KCN exhibited inhibitory activity against VIM-1–like MBLs, restoring imipenem susceptibility in *E. coli* at very low concentrations (0.04–19.55 µM). Thus, certain efflux pump inhibitors, as well as the gaseous biological mediator cyanide, have a novel action as inhibitors of MBL enzymatic activity, which, in turn, renders them potentially useful as adjunct antibacterial agents.

## Introduction

Metallo-β-lactamase (MBL)–producing Enterobacterales represent a major public health threat. They are responsible for difficult-to-treat infections and are often associated with therapeutic failure, prolonged hospitalizations and increased healthcare costs.^1^ Moreover, there has been a stagnation in the development of new clinically available antibacterial agents active against MBL–producing species. The most recent combination, aztreonam–avibactam, remains effective because aztreonam is not hydrolysed by MBLs.

MBLs contain two zinc ions (Zn^2+^) in their active site and belong to Ambler Class B.^2^ They are further subclassified into Subclasses B1, B2 and B3.^3^ Members of Subclass B1 (including IMP, NDM, VIM, SIM and SPM types) are clinically important and frequently plasmid mediated, which contributes to their global dissemination.^4^ Among these, VIM-like enzymes represent the second most prevalent group of acquired MBLs. The main members, VIM-1 (Subgroup 1) and VIM-2 (Subgroup 2), share approximately 90% amino acid identity but differ slightly in their active sites.^3,4^

Currently, there is a lack of effective therapeutic options for MBL producers. Approved β-lactamase inhibitors, such as avibactam, vaborbactam and relebactam, when combined with β-lactams, are active against serine carbapenemases but not MBLs.^5^ Combinations using newer inhibitors, such as zidebactam, nacubactam, taniborbactam or xeruborbactam, show promise against MBL producers. Other MBL inhibitors, including bismuth compounds, dimercaptosuccinic acid, bisthiazolidines, dipicolinic acid derivatives and aspergillomarasmine A, have been investigated, but none have yet been approved so far for clinical use.^6,7^ Another potential treatment is the novel siderophore cephalosporin cefiderocol; however, resistance among MBL–producing strains has already been reported.^8^

Given that overexpression of efflux pumps is a major mechanism of acquired antibiotic resistance, efflux pump inhibitors have been shown to potentiate antibiotic activity *in vitro* and *in vivo* infection models.^9^ In eukaryotic cells, carbonyl cyanide m-chlorophenylhydrazone (CCCP) and carbonyl cyanide 4-trifluoromethoxyphenylhydrazone (FCCP) act as uncouplers of oxidative phosphorylation by disrupting the ionic gradient across bacterial membranes. They indirectly inhibit efflux pumps by collapsing the proton motive force across biological membranes (as a protonophore).^10,11^ The inner mitochondrial membrane relies on a proton gradient to generate ATP via oxidative phosphorylation. CCCP and FCCP facilitate proton leakage across the membrane, thereby uncoupling the proton gradient from ATP synthesis.^12^

For the current project, our hypothesis is that CCCP, FCCP and cyanide [generated from potassium cyanide (KCN)] may have an additional mode of action. We hypothesized that they may use their negatively charged cyanide groups to bind to the positively charged zinc ions in the active site of MBLs, thereby disrupting enzyme activity and potentially restoring susceptibility of carbapenems.

## Materials and methods

### Bacterial strains

A series of isogenic *Escherichia coli* TOP10 strains carrying MBL genes on the shuttle vector pUCP24—including *bla_NDM-like_*, *bla_VIM-like_*, *bla_IMP-like_*, *blaSPM-1*, *bla_DIM-1_*, *bla_AIM-1_*, *bla_GIM-1_*, *bla_PFM-1_* and *bla_SIM-1_*, as well as Class A (*bla_KPC-2_*) and Class D (*bla_OXA-181_*) controls—were included in the study.^13,14^ A second isogenic background of *E. coli*, namely MG1655 strain, was also constructed for the study purpose carrying *bla_NDM-like_*, *bla_VIM-like_* and *bla_IMP-like_* on the same plasmid vector. And finally, *E. coli* clinical strains producing *bla_NDM-like_*, *bla_VIM-like_* and *bla_IMP-like_* were also included in the study.

### Antimicrobial susceptibility testing and checkerboard assays

Minimum inhibitory concentrations (MICs) were determined by broth microdilution using cation-adjusted Mueller–Hinton broth (CA-MHB; Bio-Rad, Marnes-la-Coquette, France) according to EUCAST guidelines.^15^ The recombinant *E. coli* isolates were tested against imipenem (IPM; HuiChem, Shanghai, China), CCCP (Sigma–Aldrich, USA), FCCP (MedChemExpress, USA) and KCN (Sigma–Aldrich). *E. coli* ATCC 25922 was used as the quality control strain.

Checkerboard assays combining IPM with CCCP (IPM/CCCP), FCCP (IPM/FCCP) and KCN (IPM/KCN) were performed to assess synergistic effects. EUCAST breakpoints for Enterobacterales were interpreted as S ≤ 2 mg/L and R > 4 mg/L.^15^ The fractional inhibitory concentration index (FICI) was calculated as follows: FICI = (MIC of Drug A in combination/MIC of Drug A alone) + (MIC of Drug B in combination/MIC of Drug B alone). Interpretation: FICI ≤ 0.5 indicates synergy; 0.5 < FICI ≤ 4, no interaction; and FICI > 4, antagonism. All assays were performed in triplicate. Additional susceptibility testing was conducted with 70 mg/L ZnSO_4_ (Sigma–Aldrich) to evaluate reversal of susceptibility.

### Determination of the 50% inhibitory concentration and enzyme purification

Crude enzyme extracts of VIM-1, VIM-4, VIM-19, VIM-83, VIM-2, IMP-1 and NDM-1 were incubated with varying concentrations of CCCP and KCN for 10 min at room temperature in 50 mM HEPES buffer (pH 7.5), 150 mM NaCl and 50 µM ZnSO_4_. Following preincubation, cephalothin (100 μM) was added, and residual hydrolytic activity was monitored at 262 nm using a Genesys 10S UV/VIS spectrophotometer (Thermo Fisher Scientific, USA). The 50% inhibitory concentration (IC_50_) was defined as the concentration reducing cephalothin hydrolysis by 50%.

The VIM-1 enzyme was additionally cloned into the pOPINF expression vector (pOPINF + VIM-1)^16^ by using the In-Fusion™ cloning kit (Takara Bio, Japan). Primers used for cloning were forward 5′ GGTCTGGAAGGTAGCGGTAGCAGTCCGTTAGCC 3′ and reverse 5′ CTGGTCTAGAAAGCCTACTCGGCGACTGAGCGATTTTT 3′. After that, pOPINF + VIM-1 was transformed into BL21-DE3 *E*. *coli* strain, and VIM-1 expression was induced with 0.5 mM/L isopropyl β-D-1-thiogalactopyranoside (IPTG; Carl Roth, Germany). VIM-1 was then extracted by sonication, and supernatant was purified by a nickel-nitrilotriacetic acid (Ni-NTA) agarose column (His GraviTrapTM GE Healthcare, USA).^17,18^ Purified proteins were analysed by SDS-PAGE. VIM-1 enzyme was then incubated with CCCP, FCCP and KCN for 10 min at room temperature in 50 mM HEPES buffer (pH 7.5), 150 mM NaCl and 50 µM ZnSO_4_. After preincubation with cephalothin, the hydrolytic activity of VIM-1 was monitored at 262 nm as described above. IC50s performed with the purified VIM-1 served as a confirmatory model for the other results obtained with crude extract. All measurements were performed in triplicate. Analysis was conducted in GraphPad Prism v9.5.1.

### Kinetic assays

Steady-state kinetic assays were performed for the purified VIM-1 enzyme, as described elsewhere.^19^ Hydrolysis of cephalothin, nitrocefin and imipenem was observed by the reduction of absorbance using SpectraMax ID5 equipment. Experiments were performed in 50 mM HEPES buffer (pH 7.5), 150 mM NaCl and 50 µM ZnSO_4_. Absorbances were measured for seven different substrate concentrations for 30 min at 25°C. Data was analysed in GraphPad Prism v9.5.1 using non-linear regression curve by Michaelis–Menten.^20^ Antibiotic coefficients are the following: cephalothin (Sigma; Δ*ɛ*_262_ = −7660 M^−1^ cm^−1^), nitrocefin (Toronto Research Chemicals, Canada; Δ*ɛ*_486_ = 15 900 M^−1^ cm^−1^) and IPM (HuiChem, China; Δ*ɛ*_297_ = 9210 M^−1^ cm^−1^). Experiments were performed in triplicate.

### Time-kill curve assays

Time-kill assays were performed as previously described only for combinations showing synergy (FICI ≤ 0.5).^21^ Briefly, recombinant strains (∼10^6^ cfu/mL) were inoculated into CA-MHB in screw cap glass tubes (to prevent loss of volatile compounds) under the following conditions: (i) no pharmacological agent (growth control), (ii–iii) IPM alone (1/4 or 1/8 MIC), (iv–v) IPM plus pharmacological agent (1/4 or 1/8 MIC + fixed chemical concentration) and (vi) pharmacological agent alone (fixed concentration). Cultures were incubated (150 rpm, 37°C) for 8 h, and aliquots were sampled at 0, 2, 4, 6 and 8 h, serially diluted and plated onto LB agar (Carl Roth, Germany) for cfu determination after 18–20 h incubation at 37°C. Experiments were performed in triplicate. Statistical analyses were conducted by calculating the area under the curve (AUC) of the cfu counting and applying a one-way analysis of variance (ANOVA) followed by Tukey’s multiple comparisons (GraphPad Prism v9.5.1).

## Results and discussion

MICs for CCCP, FCCP and KCN were 16–32, 16–32 and 16 mg/L, respectively, for all strains producing various MBLs or serine carbapenemases (Classes A and D) (Table 1). Checkerboard assays were performed using CCCP [0.05–19.55 µM (0.01–4 mg/L)], FCCP [0.04–15.74 µM (0.01–4 mg/L)] and KCN [0.15–15.36 µM (0.01–1 mg/L)] in combination with IPM. Synergy was observed in strains producing VIM-1, VIM-4, VIM-19 and VIM-83 at one or more tested concentrations. In TOP10 strains carrying VIM-1 and VIM-4, synergy was observed for all combinations tested, whereas VIM-19 lacked synergy only for IPM/KCN at 0.05 µM, and VIM-83 lacked synergy for IPM/CCCP at 4.89 µM and IPM/KCN at 0.05 µM. Among the MG1655 derivatives, synergy was observed only for the VIM-1 producer for CCCP/IPM at 19.55 µM and FCCP/IPM at 15.74 µM. The same concentrations were synergic in the clinical strain C2-VIM-1. In contrast for the C3-VIM-4 and C4-VIM-4, synergy was observed for CCCP/IPM at 4.89, 9.77 and 19.55 µM and for FCCP/IPM at 3.93, 7.87 and 15.74 µM). No synergy was observed in any other clinical strain.

**Table 1.** MICs and FIC indexes of *E. coli* recombinant and clinical strains producing different carbapenemases in the presence of CCCP, FCCP and KCN at various concentrations

Strains*		MICs (mg/L) and FICI												
		IPM	IPM/CCCP^a^	FICI	IPM/CCCP^b^	FICI	IPM/FCCP^c^	FICI	IPM/FCCP^d^	FICI	IPM/KCN^e^	FICI	IPM/KCN^f^	FICI
	TOP10	0.06	0.06	1.00	0.06	1.06	0.06	1.00	0.06	1.03	0.06	1.00	0.06	1.06
	TOP10 + pucp24	0.06	0.06	1.00	0.06	1.06	0.06	1.00	0.06	1.03	0.06	1.00	0.06	1.06
	TOP10 + pucp24 + NDM − 1	8	8	1.00	8	1.06	8	1.00	8	1.03	8	1.00	8	1.06
	TOP10 + pucp24 + NDM − 5	4	4	1.00	4	1.06	4	1.00	4	1.03	4	1.00	4	1.06
	TOP10 + pucp24 + NDM − 7	4	4	1.00	4	1.06	4	1.00	4	1.03	4	1.00	4	1.06
	TOP10 + pucp24 + NDM − 9	8	8	1.00	8	1.06	8	1.00	8	1.03	8	1.00	8	1.06
	TOP10 + pucp24 + NDM − 19	8	8	1.00	8	1.06	8	1.00	8	1.03	8	1.00	8	1.06
	TOP10 + pucp24 + VIM − 1	4	1	**0**.**25**	1	**0**.**31**	1	**0**.**25**	1	**0**.**28**	1	**0**.**25**	1	**0**.**31**
	TOP10 + pucp24 + VIM − 2	1	1	1.00	1	1.06	1	1.00	1	1.03	1	1.00	1	1.06
	TOP10 + pucp24 + VIM − 4	4	1	**0**.**25**	1	**0**.**31**	1	**0**.**25**	1	**0**.**28**	1	**0**.**25**	1	**0**.**31**
	TOP10 + pucp24 + VIM − 19	8	2	**0**.**25**	2	**0**.**31**	2	**0**.**25**	2	**0**.**28**	4	0.56	2	**0**.**31**
	TOP10 + pucp24 + VIM − 36	4	4	1.00	4	1.06	4	1.00	4	1.03	4	1.00	4	1.06
	TOP10 + pucp24 + VIM − 83	4	1	**0**.**25**	2	0.56	1	**0**.**25**	1	**0**.**28**	4	1.00	1	**0**.**31**
	TOP10 + pucp24 + IMP − 1	4	4	1.00	4	1.06	4	1.00	4	1.03	4	1.00	4	1.06
	TOP10 + pucp24 + IMP − 10	4	4	1.00	4	1.06	4	1.00	4	1.03	4	1.00	4	1.06
	TOP10 + pucp24 + IMP − 11	4	4	1.00	4	1.06	4	1.00	4	1.03	4	1.00	4	1.06
	TOP10 + pucp24 + IMP − 13	4	4	1.00	4	1.06	4	1.00	4	1.03	4	1.00	4	1.06
	TOP10 + pucp24 + SPM − 1	1	1	1.00	1	1.06	1	1.00	1	1.03	1	1.00	1	1.06
	TOP10 + pucp24 + DIM − 1	1	1	1.00	1	1.06	1	1.00	1	1.03	1	1.00	1	1.06
	TOP10 + pucp24 + AIM − 1	1	1	1.00	1	1.06	1	1.00	1	1.03	1	1.00	1	1.06
	TOP10 + pucp24 + GIM − 1	1	1	1.00	1	1.06	1	1.00	1	1.03	1	1.00	1	1.06
	TOP10 + pucp24 + PFM − 1	1	1	1.00	1	1.06	1	1.00	1	1.03	1	1.00	1	1.06
	TOP10 + pucp24 + SIM − 1	1	1	1.00	1	1.06	1	1.00	1	1.03	1	1.00	1	1.06
	TOP10 + pucp24 + KPC − 2	16	16	1.00	16	1.06	16	1.00	16	1.03	16	1.00	32	2.06
	TOP10 + pucp24 + OXA − 181	0.5	0.5	1.00	0.5	1.06	0.5	1.00	0.5	1.03	0.5	1.00	0.5	1.06

MG1655^#^		IPM	IPM/CCCP^b^	FICI	IPM/CCCP^g^	FICI	IPM/CCCP^h^	FICI	IPM/FCCP^d^	FICI	IPM/FCCP^i^	FICI	IPM/FCCP^j^	FICI
	MG1655	0.12	0.12	1.03	0.12	1.06	0.12	1.13	0.12	1.03	0.12	1.06	0.12	1.13
	MG1655 + pucp24 + NDM − 1	64	64	1.03	64	1.06	64	1.13	64	1.03	64	1.06	64	1.13
	MG1655 + pucp24 + VIM − 1	8	4	0.53	4	0.56	2	**0**.**38**	4	0.53	4	0.56	4	**0**.**38**
	MG1655 + pucp24 + VIM − 2	1	1	1.03	1	1.06	1	1.13	1	1.03	1	1.06	1	1.13
	MG1655 + pucp24 + IMP − 1	2	2	1.06	2	1.13	2	1.25	2	1.06	2	1.13	2	1.25

Clinical strains^#^		IPM	IPM/CCCP^b^	FICI	IPM/CCCP^g^	FICI	IPM/CCCP^h^	FICI	IPM/FCCP^d^	FICI	IPM/FCCP^i^	FICI	IPM/FCCP^j^	FICI
	C1 − NDM − 1	16	16	1.06	16	1.13	16	1.25	16	1.02	16	1.03	16	1.06
	C2 − NDM − 1	16	16	1.06	16	1.13	16	1.25	16	1.03	16	1.06	16	1.13
	C3 − VIM − 1	2	2	1.06	2	1.13	1	0.75	2	1.03	1	0.56	1	0.63
	C4 − VIM − 1	8	4	0.53	4	0.56	2	**0**.**38**	4	0.53	4	0.56	2	**0**.**38**
	C5 − VIM − 4	16	4	**0**.**31**	4	**0**.**38**	4	**0**.**50**	4	**0**.**28**	4	**0**.**31**	4	**0**.**38**
	C6 − VIM − 4	32	8	**0**.**31**	8	**0**.**38**	4	**0**.**38**	4	**0**.**16**	4	**0**.**19**	4	**0**.**25**
	C7 − IMP − 1	2	2	1.06	2	1.13	2	1.25	2	1.06	2	1.13	2	1.25
	C8 − IMP − 1	4	4	1.06	4	1.13	4	1.25	4	1.06	4	1.13	4	1.25

IPM, imipenem; CCCP, carbonyl cyanide m-chlorophenylhydrazone; FCCP, carbonyl cyanide 4-trifluoromethoxyphenylhydrazone; KCN, potassium cyanide.

Underline indicates significant reductions (≥2-fold) from the initial IPM MIC; bold indicates synergistic FICIs (≤0.5).

^a^CCCP: 0.05 µM; ^b^CCCP: 4.89 µM; ^c^FCCP: 0.04 µM; ^d^FCCP: 3.93 µM; ^e^KCN: 0.15 µM; ^f^KCN: 15.36 µM; ^g^CCCP: 9.77 µM; ^h^19.55 µM; ^i^7.87 µM; ^j^15.74 µM.

^*^, The MICs for CCCP, FCCP and KCN were 16–32 mg/L, 16–32 mg/L and 16 mg/L, respectively, for all the strains.

^#^, No synergy effect was observed when combining different concentrations of KCN with IPM for the *E. coli* recombinant MG1655 and clinical strains; range of CCCP and FCCP was adapted.

Notably, CCCP, FCCP and KCN exhibit activity against *E. coli* and that their known role as proton gradient uncouplers and respiratory inhibitors may partially impair efflux pump function. However, if the observed effect were solely due to efflux pump inhibition potentiating IPM, a similar synergistic effect would be expected across *all E. coli* strains tested, particularly the isogenic TOP10 and MG1655 backgrounds, regardless of the carbapenemase produced. Instead, synergy was selectively observed in VIM-1 producers. These enzymes are MBLS with zinc-dependent catalytic mechanisms with a specific catalytic site that is potentially susceptible to inhibition via chelation or displacement of zinc ions by cyanide-containing molecules.

Our findings support this hypothesis by demonstrating a strong and selective effect of CCCP, FCCP and KCN on VIM-1–like enzymes with no synergy observed for other MBLs, including VIM-2-like (VIM-2, VIM-36), IMP-1 or NDM-1.

Since dipicolinic acid is a known zinc chelator,^22^ and because CN is also known to bind to zinc moieties in proteins,^23,24^ we hypothesized that the tested compounds may chelate or displace Zn^2+^ ions from the active site of VIM-1–like MBLs (Figure S1, available as Supplementary data at *JAC* Online).^25^ This hypothesis was confirmed experimentally by obtaining a reversed susceptibility upon addition of ZnSO_4_ with increasing MICs from 1 to 16 mg/L (Table S1).

IC_50_ and time-kill assays were performed for CCCP and KCN (FCCP was only used for IC_50_ determination with the purified VIM-1). IC_50_ values confirmed selective inhibition of VIM-1–like enzymes (VIM-1, VIM-4, VIM-19, VIM-83) compared to VIM-2, IMP-1 and NDM-1 (Table S2). The lowest IC_50_ values were 0.2 mM for CCCP (VIM-83) and 1.7 mM for KCN (VIM-83), whereas values exceeded 30 mM for IMP-1 and VIM-2 producers. Using purified VIM-1, IC_50_ values were 0.2 mM for CCCP, 2.8 mM for KCN and 0.1 mM for FCCP, consistent with results obtained for crude extracts.

**Figure 1.**
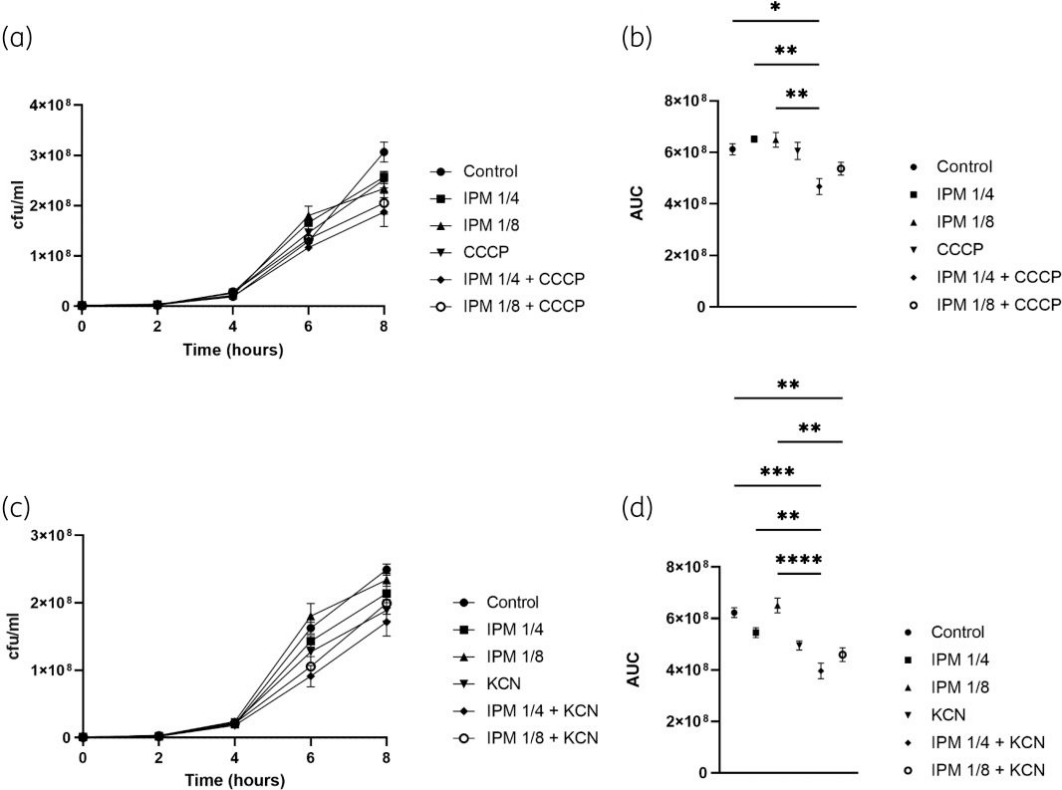
Time-kill assays for *E. coli* TOP 10 + pUCP24 + VIM-1 recombinant strain. (a) Time-kill curve in the presence or absence of CCCP. (b) AUC in the presence or absence of CCCP. (c) Time-kill curve in the presence or absence of KCN. (d) AUC in the presence or absence of KCN. IPM concentration used was 1/4 (1 mg/L) or 1/8 (0.5 mg/L) of the MIC. CCCP fixed concentration used was 0.05 µM. KCN fixed concentration used was 15.36 µM. Data is shown as mean ± SEM. *, *P*-value ≤ 0.05; **, *P*-value ≤ 0.01; ***, *P*-value ≤ 0.001; ****, *P*-value ≤ 0.0001.

In time-kill assays with a VIM-1–producing strain, the combination of IPM (1/4 MIC) with CCCP (0.05 µM) reduced cfu counts by 1.3–1.4-fold compared with controls and IPM alone. Similar reductions were observed for IPM (1/4 MIC) plus KCN (15.36 µM) and for IPM (1/8 MIC) plus KCN (15.36 µM) (Figure 1). Kinetic assays with the VIM-1 enzyme at 10 nM concentration yielded kcat/Km values (µM^−1^ s^−1^) of 3.4 × 10^4^ or cephalothin, 1.1 × 10^6^ for nitrocefin and 5.3  ×  10^4^, for IPM (Table S3).

Importantly, FCCP and KCN have not previously been reported to restore antibiotic susceptibility. The concentrations used here were non-toxic and below those typically used for efflux inhibition: CCCP (0.05 µM) and FCCP (0.04 µM) are much lower than the standard 35–40 µM used *in vitro* and 1–12 µM used in eukaryotic cell assays.^26^ KCN was active in isogenic VIM-1–like TOP10 producers at 0.15–15.36 µM, well below the concentrations of cyanide where it exerts cytotoxic effects in mammalian cells in culture (800 μM, equivalent to 50 mg), and in fact, sub-micromolar concentrations of cyanide can exert protective and bioenergetic stimulatory effects in mammalian cells.^23,27–29^ A limitation of this study is that we could not elucidate the mechanistic basis for the strong selectivity of CCCP, FCCP and KCN towards VIM-1–like enzymes compared to other MBLs. Similar selective effects have been reported for many of the MBLs in development, such as taniborbactam or xeruborbactam. Such selective effects do not limit their industrial development. In addition to the potential translational implications of these findings, our observations also highlight a methodological caveat: *in vitro* studies using cyanide-containing efflux inhibitors may be confounded when testing bacteria expressing MBLs.

### Conclusion

This study provides the first proof of concept that cyanide-containing efflux inhibitors, and cyanide-based molecules more broadly, can inhibit certain MBLs and restore susceptibility to carbapenems. The exposed cyanide group may chelate or displace zinc ions in the MBL active site (Figure S1).^25^ Efflux pump inhibitors, such as CCCP, FCCP and KCN, therefore display dual activity: efflux inhibition and MBL inhibition. These findings highlight their potential as adjuvant agents for the treatment of infections caused by VIM-1–like producers. Further *in vivo* studies are warranted to evaluate their therapeutic potential in animal infection models.

## Supplementary Material

dkag122_Supplementary_Data

## Supplementary data

Figure S1 and Tables S1, S2 and S3 are available as Supplementary data at *JAC* Online.
