# Supplementary material for: Metallo-β-lactamase–producing Enterobacterales: cyanide-containing efflux pump inhibitors as potential dual-activity inhibitors
Source: J Antimicrob Chemother. 2026 Mar 31;81(4):dkag122. doi: 10.1093/jac/dkag122 (PMC13036317; doi:10.1093/jac/dkag122)
Supplement: dkag122_Supplementary_Data [file dkag122_supplementary_data.docx]

**Supplementary Table S1.** MICs demonstrating recovery of resistance upon addition of zinc for recombinant strains carrying VIM-1 and VIM-4 enzymes.

| **Strains** | **Compounds (mg/L)** | | | | | | |
| --- | --- | --- | --- | --- | --- | --- | --- |
|  | **IPM** | **IPM + CCCP** | **IPM + CCCP + ZnSO4** | **IPM + FCCP** | **IPM + FCCP + ZnSO4** | **IPM + KCN** | **IPM + KCN + ZnSO4** |
| TOP10 | 0.06 | 0.06 | 0.06 | 0.06 | 0.06 | 0.06 | 0.06 |
| TOP10+pucp24 | 0.06 | 0.06 | 0.06 | 0.06 | 0.06 | 0.06 | 0.06 |
| TOP10+pucp24+VIM-1 | 4 | 1 | **16** | 1 | **16** | 1 | **16** |
| TOP10+pucp24+VIM-4 | 4 | 1 | **16** | 1 | **16** | 1 | **16** |
| MG1655 | 0.12 | 0.12 | 0.12 | 0.12 | 0.12 | 0.12 | 0.12 |
| MG1655+pucp24+VIM-1 | 8 | 2 | **8** | 4 | **8** | NA | NA |
| C3-VIM-1 | 2 | 1 | 2 | 1 | 2 | NA | NA |
| C4-VIM-1 | 8 | 2 | **8** | 2 | **8** | NA | NA |
| C5-VIM-4 | 16 | 4 | **16** | 4 | **16** | NA | NA |
| C6-VIM-4 | 32 | 4 | **32** | 4 | **32** | NA | NA |

Fixed concentration of CCCP, FCCP and KCN for TOP10 recombinant strains were 0.05 µM, 0.04 µM and 15.36 µM, respectively. Fixed concentration of CCCP and FCCP for MG1655 recombinant strain and clinical strains were 19.55 µM and 15.74 µM, respectively. ZnSO_4_ fixed concentration used was 70 mg/L. Underlined number stand for significant reductions (≥ 2-fold) as compared to the initial IPM MIC. Boldened numbers indicate significant increases (≥ 2-fold) as compared to the MIC values of the combinations IPM plus CCCP, FCCP, and KCN. NA, not available.

**Supplementary Table S2**. Inhibitory concentrations of CCCP and KCN against crude extracts of VIM-1, VIM-4, VIM-19, VIM-83, VIM-2, IMP-1, NDM-1 and CCCP, KCN, and FCCP against purified VIM-1 enzyme at 10 nM.

| **Enzyme** | **IC50 (mM)** | | | | | |
| --- | --- | --- | --- | --- | --- | --- |
|  | **Crude extracts** | | **Purified enzyme** | | | |
|  | **CCCP** | **KCN** | | **CCCP** | **KCN** | **FCCP** |
| VIM-1 | 0.4 | 3.2 | | 0.2 | 2.8 | 0.1 |
| VIM-4 | 0.4 | 4.4 | |  |  |  |
| VIM-19 | 0.8 | 6.6 | |  |  |  |
| VIM-83 | 0.2 | 1.7 | |  |  |  |
| VIM-2 | 16.2 | > 30 | |  |  |  |
| IMP-1 | 17.6 | > 30 | |  |  |  |
| NDM-1 | 11.9 | 19.5 | |  |  |  |

**Supplementary Table S3.** Kinetic parameters of VIM-1 at 10 nM against cephalothin, nitrocefin, and imipenem.

| **Antibiotic** | **Kcat (s^-1^)** | **Km (µM)** | **Kcat/Km (µM^-1^ s^-1^)** |
| --- | --- | --- | --- |
| Cephalothin | 5 | 139 | 3.4 x 10^4^ |
| Nitrocefin | 76 | 13 | 1.1 x 10^6^ |
| Imipenem | 11 | 217 | 5.3 x 10^4^ |


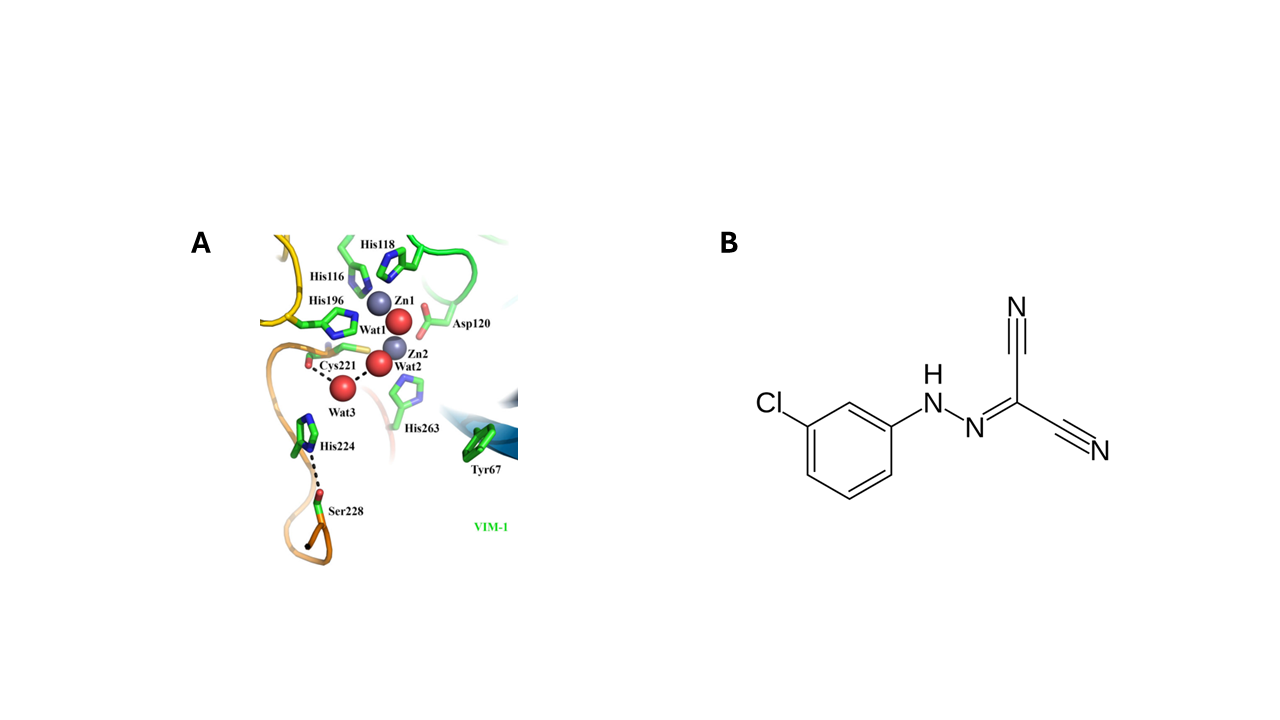


**Figure S1**. Hypothesis binding of the cyanide group and displacement of the zinc ions from VIM-1 active site. **A**. Active site of VIM-1 MBL showing the two zinc molecules (blue balls) and three water molecules (red balls). **B**. Structure of Carbonyl Cyanide m-Chlorophenylhydrazone (CCCP). Hydrogen-bonding interactions are depicted as dashed lines. The cyanide moiety of CCCP or FCCP or cyanide itself (released from KCN) are proposed to displace zinc ions from the VIM-1 active site. This figure is adapted from Salimraj et al.^25^
